# Diaphragm ultrasonography: A picture is worth a thousand words

**DOI:** 10.1113/EP092452

**Published:** 2024-12-06

**Authors:** Ken D. O'Halloran

**Affiliations:** ^1^ Department of Physiology University College Cork Cork Ireland

**Keywords:** diaphragm, fatigue, transdiaphragmatic pressure, ultrasound

1

The diaphragm is the primary muscle of inspiration. Diaphragm dysfunction is surprisingly common, presenting in acute critical care, cardiorespiratory diseases, cancer cachexia, endocrine disorders, and neuromuscular and neurodegenerative diseases amongst other scenarios (Laghi et al., 2021). Assessment of diaphragm performance involves measurement of transdiaphragmatic pressure, which assesses the force‐generating capacity of the diaphragm in situ, typically by eliciting a twitch contraction of the diaphragm by electrical or magnetic activation of the phrenic nerves. A limitation of the approach, however, is that it does not reveal explicit detail of the shortening capacity of the muscle during contraction, which is an important aspect of diaphragm performance. The dome‐shaped diaphragm muscle flattens during contraction increasing intra‐thoracic volume, thereby decreasing intra‐thoracic pressure, facilitating airflow into the lungs during inspiration. Work (force × distance) and power (force × velocity) are key measures of muscle performance, and therefore tools to complement the assessment of diaphragm force are warranted and could have additional experimental and clinical value. In this issue of *Experimental Physiology*, Illidi and Romer (2025) combine ultrasonography and respiratory manometry to quantify the change in contractile performance of the diaphragm during fatigue in healthy humans.

Recreationally active, young male participants were recruited and enrolled in randomised fashion to two commonly employed experimental protocols designed to elicit diaphragm fatigue: (1) a low‐force, high‐velocity task of maximal isocapnic ventilation, and (2) a high‐force, low‐velocity task of maximal inspiratory resistive loading. Transdiaphragmatic pressure (gastric pressure minus oesophageal pressure) and ultrasound‐derived data (diaphragm excursion) in response to phrenic nerve stimulation by magnetic stimulation and during a CO_2_‐rebreathe test were performed before and after each fatiguing task. Two post‐trial time points were assessed to measure fatigue at 10 and 30 min following each loading task. Measurement of diaphragm excursion parameters allowed for the calculation of mean excursion velocity. Diaphragm work and power were subsequently calculated incorporating pressure and ultrasound‐derived measurements.

Muscle fatigue is the reversible decline in force or power due to contractile activity. Based upon the measurement of transdiaphragmatic pressure, both loading tasks used in the study elicited diaphragm fatigue with similar reductions in transdiaphragmatic twitch pressure for maximal isocapnic ventilation and inspiratory resistive loading tasks. These reductions were notably less than those reported by others for the same tasks (Luo et al., 2001), and reductions were not seen in all participants, which the authors surmise may relate to participant demographics and in particular training status, as several participants in the study by Illidi and Romer (2025) were competitive athletes.

Ultrasonography proved feasible for the assessment of fatigue‐induced changes in diaphragm contraction in healthy participants. Inspiratory resistive loading resulted in significant decreases in ultrasound‐derived measures of crural diaphragm absolute shortening and shortening velocity. The dominant change in diaphragm excursion measures, especially velocity of shortening, combined with the secondary changes in transdiaphragmatic pressure resulted in significant decreases in calculated diaphragm work and power lasting at least 30 min following completion of the loading task. Surprisingly, but intriguingly, maximal isocapnic ventilation had no effect on any of the ultrasound‐derived measures. The authors offer a fair‐spirited discussion addressing this unexpected outcome. In short, it is complicated! A variety of inter‐related factors contribute to fatigue under different loading conditions. Whereas differences in the magnitude of fatigue elicited by force/pressure tasks compared to velocity/flow tasks have been described by other investigators, previous assessments of contractile function used maximal inspiratory manoeuvres that are highly dependent upon participant effort and motivation, which further complicates interpretation. A key advantage of the assessment of fatigue employed by Illidi and Romer (2025) is the use of objective, effort‐independent, combined multi‐metric determination of contractile performance.

Illidi and Romer (2025) confirm the benefit of attendant use of ultrasound to assess diaphragm function demonstrating the power of imaging. But as always, the devil is in the detail! The study design and execution were meticulous. Yet, a relatively small sample size of young fit males was studied, including athletes, which limits generalisability of the findings to a wider population, especially an older population, which may be especially relevant to the consideration of diaphragm performance given the recognised decline in diaphragm and respiratory performance with advancing age. By design the study was restricted to male participants owing to the known sex differences in fatigue (Guenette et al., 2010), but diaphragm dysfunction in clinical settings is indiscriminate, so it will be important into the future to build a comparable portfolio in female participants. Combined diaphragm ultrasonography and respiratory manometry may have important application in the context of critical care, to monitor the expected diaphragm dysfunction following prolonged mechanical ventilation and to complement existing approaches to successful weaning, avoiding extubation failure (Dres et al., 2017). To date, diaphragm ultrasound alone to predict weaning outcome has yielded inconsistent results (see Laghi et al., 2021).

A potential limitation of the study by Illidi and Romer (2025) is inference of diaphragm muscle performance based on crural diaphragm excursions. Whereas the action of the crural diaphragm is generally reflective of whole muscle, the costal diaphragm compartments contribute mostly to the action of the diaphragm as an inspiratory pump. Moreover, generally with ultrasound assessments, there are concerns with repeatability and reproducibility (i.e., variation in repeated measures within and between sonographers). Nevertheless, Illidi and Romer (2025) provide us with a potentially valuable tool to interrogate diaphragm function in health and disease.

## AUTHOR CONTRIBUTIONS

Sole author.

## CONFLICT OF INTEREST

The author declares no conflicts of interest.

## FUNDING INFORMATION

None.
